# Bidirectional Relationships among Children's Perceived Competence, Motor Skill Competence, Physical Activity, and Cardiorespiratory Fitness across One School Year

**DOI:** 10.1155/2021/1704947

**Published:** 2021-08-23

**Authors:** Suryeon Ryu, Jung Eun Lee, Nan Zeng, David Stodden, Daniel J. McDonough, Wenxi Liu, Zan Gao

**Affiliations:** ^1^School of Kinesiology, University of Minnesota-Twin Cities, Minneapolis, MN 55455, USA; ^2^Department of Applied Human Sciences, University of Minnesota, Duluth, MN, USA; ^3^Prevention Research Center, Department of Pediatrics, University of New Mexico Health Sciences Center, Albuquerque, NM, USA; ^4^College of Education, University of South Carolina, Columbia, SC, USA

## Abstract

**Purpose:**

The bidirectional associations among children's motor skill competence (MSC), perceived competence (PC), physical activity (PA), and cardiorespiratory fitness (CRF) over time remain unanswered. This study is aimed at discerning the bidirectional relationships among elementary school children's MSC, PC, PA and, CRF over the course of one school year.

**Methods:**

A total of 261 second and third grade children (127 boys, 134 girls; mean_age_ = 8.27 years; BMI = 18.22 ± 3.71) were recruited from two Texas elementary schools. Approximately 73.56% of participants were White American. Children's baseline data were assessed in September/October in 2012 (Time1), and identical assessments were conducted in April/May in 2013 (Time2). MSC was assessed using product-oriented skill tests (e.g., throw, kick, and jump). PC was assessed via the Pictorial Scale of Perceived Competence for Children. Minutes spent in moderate-to-vigorous PA (MVPA) was assessed using ActiGraph GT3X+ accelerometers for five days, and CRF was assessed by the PACER test. Six age- and body mass index-adjusted cross-lagged panel models were used to test the relationships between the variables.

**Results:**

We observed that T1 MSC significantly predicted T2 MSC (*β* = 0.59; *p* < 0.01), T2 CRF (*β* = 0.28; *p* < 0.01), and T2 MVPA (*β* = 0.18; *p* < 0.01). Children's CRF was a positive predictor for T2 CRF (*β* = 0.56; *p* < 0.01) and T2 MSC (*β* = 0.13; *p* < 0.05) Additionally, T1 MVPA significantly predicted T2 MVPA (*β* = 0.30; *p* < 0.01) and T2 PC (*β* = −0.14; *p* < 0.05).

**Conclusion:**

Findings suggested a fully bidirectional relationship between elementary children's MSC and CRF. Other bidirectional relationships among the variables were only partially supported. Educators and health professionals need to emphasize the importance of developing both MSC and CRF to maintain physical health over time.

## 1. Introduction

Regular participation in physical activity (PA) has been linked to improved physical health (e.g., cardiorespiratory fitness), decreased chronic disease rates (e.g., diabetes), and improved psychological wellbeing (e.g., perceived competence) in children and adolescents [1, 2]. Consequently, promoting daily PA participation in school-aged children has become an important public health initiative. While many studies have focused on increasing children's PA participation [3], better understanding the link between multiple health-related physical and psychological constructs and how they collectively impact trajectories of movement development are needed [4].

As known, the development of motor skills has been positively associated with PA and cardiorespiratory fitness [5, 6]. Physical perceived competence (PC) refers to children's self-evaluative judgments about their ability to perform a certain task or learn a certain behavior [7]. Barnett et al. demonstrated that children's motor skill competence (MSC) and PC had mutual effects on their PA and fitness. [5] Additionally, Vedul-Kjelsås et al. found that both fitness and MSC were significantly correlated to PC and yielded gender difference in the relationships as well [6].

According to Competence Motivation Theory, a child's behavior is driven by intrinsic engagement in mastery experiences (i.e., MSC) and is supported by feedback from both internal and external sources, which in turn influences how a child perceives his/her skills [8]. Interestingly, PC in early and into middle childhood is more driven by external feedback, and thus, PC is inflated in many children and generally demonstrates weak relationships with MSC. In general, high levels of PC are associated with increased PA participation, which promotes further engagement in MSC and related PA behaviors [8]. However, MSC needs to be acquired through context-specific practice and experiences as they do not develop naturally [9]. Consequently, increased exposure to developmentally appropriate PA experiences would enhance MSC and PA both acutely [10, 11], as well as longitudianlly [12]. The development of locomotor and object projection skills in childhood also influences cardiorespiratory fitness both directly and indirectly as continued effortful practice and performance of skills, and their application in multiple types of activities directly requires high neuromuscular demand (e.g., high levels of eccentric/concentric muscle activation) [13–15] and continued metabolic demand [10]. Thus, associations among MSC, PC, CRF, and PA are suggested to strengthen children across childhood and into adolescence [9]. However, limited research has examined how relationships among these respective physical and psychological variables change across time and which variables seem to be the driving factors in these dynamic relationships [16–18].

The purpose of this study, therefore, was to examine bidirectional relationships among elementary school children's MSC, PC, PA, and CRF over the course of one school year. We hypothesized that there would be positive bidirectional relationships between (1) MSC and CRF, (2) MSC and MVPA, (3) MSC and PC, (4) MVPA and CRF, (5) CRF and PC, and (6) PC and MVPA. To our knowledge, this is the first study investigating dynamic bidirectional relationships of these variables using cross-lagged panel models. Examining bidirectional relationships of children's MSC, PC, PA, and CRF will help researchers and health professionals to better understand how effective PA and health promotion strategies may be implemented for elementary school children.

## 2. Methods

### 2.1. Participants and the Research Setting

This study was a secondary data analysis paper from a larger parent trial, which was completed in the state of Texas of the United States, 2015 [19]. Participants were 261 second and third grade children (127 boys, 134 girls; mean_age_ = 8.27, SD = 0.70, BMI = 18.22 ± 3.71) from two Title I (i.e., >50% of students were from low income families and received free or reduced-price meal at school) elementary schools. The ethnicity breakdown was as follows: 192 White Americans (including Latino White and non-Latino White), 42 Native Hawaiians or Other Pacific Islanders, 16 Native Americans, 7 African Americans, and 4 Asian Americans. Both schools were similar in light of teacher quality, overall school curricula, and socioeconomic status. In other words, both schools required 125 minutes of weekly PA programs (physical education or physical education plus active video games) at schools. They also had a recess and some extra recess time at lunch. The inclusion criteria for this study were as follows: (1) enrolled in a public elementary school, (2) children aged 7-9 years, (3) without a diagnosed physical or mental disability according to school records, and (4) provision of parental consent and child assent. The inclusion criteria were verified via the school records and self-reported demographic survey. The study was approved by the University Institutional Review Board, and both parental consent and child assent were obtained prior to the study.

### 2.2. Procedures

Children's MVPA, MSC, cardiorespiratory fitness (CRF), PC, and BMI were measured in September/October 2012 and again in April/May 2013. Informed parental consent forms and child assent forms were obtained in the first week of the school year in accordance with the University Institutional Review Board and school district requirements. Participants' baseline MVPA, CRF, MSC, and PC were measured by the researchers during the first month of the school year. All participants also underwent assessments of their MVPA, CRF, MSC, and PC by the same research staff at the end of the school year (approximately 8 months later).

### 2.3. Instruments

Demographic data were measured through self-reported demographic questionnaire, except for BMI and body fat percentage. Children's body fat percentage was calculated with predetermined formulas based on the measures of triceps and calf via skinfold calipers. Their height and weight (to calculate BMI) were also assessed by valid instruments.

#### 2.3.1. MSC

The researchers assessed children's MSC data from the product scores from 5 motor skills during scheduled physical education classes. Product scores (run, kick, and throw speed and hop and jump distance) are sensitive discriminators of MSC. High product scores are associated with more advanced developmental levels of MSC [20, 21] and with higher levels of fitness [22]. Run speed was measured with a 30-foot run (allowing for 10 feet of acceleration) on a level surface. Running was videotaped to digitize (Dartfish Inc.) the maximum distance covered in 2 stride cycles/unit time. Maximum kick and throw maximum speed measures multipoint object-projection skill [23]. The ball speeds were measured with a radar gun [13]. A tennis ball was thrown, and a playground ball was kicked with maximum effort from 30 feet [23]. Standing long jump distance was measured in cm [23]. Hopping was videotaped to digitize the average hop stride length in cm (from heel to heel) for 4 consecutive hop cycles of each leg. Jump and hop data were normalized to a percent of standing height [24, 25]. All these measures of MSC have been validated among elementary school children. They were evaluated in the elementary school gymnasium during the time allocated to physical education. The mean of standardize scores of those product scores was used as the MSC outcome.

#### 2.3.2. Perceived Competence

Six questions relating to perceived physical competence from the Pictorial Scale of Perceived Competence and Social Acceptance (PSPCSA) [26, 27] were selected to examine PC. The subscale was selected for the following reasons: (1) it has strong psychometric properties for children 7 years and older (e.g., *α* > 0.70) [26–28], (2) it is developmental in nature reflecting children's changing perception of self [27, 29], and (3) it is widely used and accepted in the literature [29]. PC was administered in small groups during physical education.

#### 2.3.3. Physical Activity Levels

Children's 5-day daily PA levels were assessed using ActiGraph GT3X+ accelerometers (Pensacola, FL). The ActiGraph is a valid and reliable measure of PA among children at school and free-living settings [19]. Participants were instructed to wear a belt with the accelerometers on the right hip during waking hours, with the exception of time spent bathing and engaging in other activities involving water. Children wore the belt, and the accelerometers for 5 full days (3 weekday and 2 weekend days) [30] and activity counts were set at a 15-second epoch. Participants' average minutes engaged in moderate to vigorous PA intensity levels per day for the 5 days was used as the outcome variables. The activity counts recorded were interpreted using empirically based cut points that define different intensities of children's PA. Acceptable inclusion criteria for the PA data were recording of an average of 10 hours of accelerometer data per day for the 5 days [31, 32]. Compliance with wearing accelerometers was facilitated according to Trost [30].

#### 2.3.4. Cardiorespiratory Fitness

The participants' CRF was measured by Progressive Aerobic Cardiovascular Endurance Run, a component of the FITNESSGRAM test to measure cardiorespiratory efficiency [33]. It was assessed and evaluated by the investigative team in the school gymnasium during the time allocated to physical education.

### 2.4. Data Analysis

All data analyses were conducted in STATA statistical software (version 15.0; StataCorp, College Station, TX, USA). This study employed repeated-measure design, and the main variables used in the analyses included MVPA, MSC, PC, and CRF for both T1 and T2. T1 age and BMI were used as the covariates. A sample of 261 at T1 and a sample of 241 of children were used in data analysis. Before the primary analyses, a paired *t* test was used to compare variable scores retained in the study across two time points of assessment. Significance level was set to 0.05 for all analyses, and effect sizes were categorized as small*d* ≤ 0.2, medium0.2 < *d* ≤ 0.5, and large0.5 < *d* ≤ 0.8 [34, 35].

The primary analyses were conducted using a structural equation modeling (SEM) framework within STATAS's SEM builder. A cross-lagged panel model using Full Information Maximum Likelihood, which used all cases within an analysis, regardless of any missing data was used to examine the dynamic relationships among the variables between two time points (i.e., baseline and 1-year follow-up). Specifically, six cross-lagged panel models were used to test the relationships between (1) MSC and CRF, (2) MSC and MVPA, (3) MSC and PC, (4) CRF and MVPA, (5) CRF and PC, and (6) MVPA and PC. Analyses of six cross-lagged panel models were run for the whole sample, girls and boys, respectively. The standardized regression coefficients (*β*-coefficients) with corresponding 95% confidence intervals (CIs) were reported in the present study. Overall model fit of data was reported as the Comparative Fit Index (CFI) and Root Mean Square Error of Approximation (RMSEA). RMSEA was considered to be excellent if <0.05, good if <0.08, and normal <0.1, and for CFI, it was determined to be excellent if ≥0.90 [36, 37]. Equation-level goodness-of-fit statistics were also computed and reported as the coefficients of determination (*R*^2^).

The strength of using a cross-lagged model panel approach is that it allows researchers to explore the autoregressive and cross-lagged pathways. The autoregressive effect identifies an association of a variable on itself at a later time, and the cross-lagged effects display an association of a variable with another variable at a later point within the model [38]. Therefore, the cross-lagged model allows for identification of possible bidirectional relationships of variables over time.

## 3. Results

Table 1 shows the descriptive statistics for all variables at both time points, stratified by gender. When comparing T1 and T2, there were significant mean differences among age (*t* = −27.14, *p* < 0.01, *d* = −2.38), MVPA (*t* = −4.55, *p* < 0.01, *d* = −0.41), PC (*t* = 10.30, *p* < 0.01, *d* = 0.92), and CRF (*t* = −5.37, *p* < 0.01, *d* = −0.48). When comparing between boys and girls, there were significant mean differences in MVPA (T1: *t* = 2.74, *p* < 0.01, *d* = .34; T2: *t* = 3.09, *p* < 0.05, *d* = 0.40), MSC (T1: *t* = 9.20, *p* < 0.01, *d* = 1.15; T2: *t* = 8.55, *p* < 0.01, *d* = 1.18), and CRF (T1: *t* = 4.48, *p* < 0.01, *d* = .56; T2: *t* = 4.37, *p* < 0.01, *d* = 0.58) at both time points with boys having greater MVPA, MSC, and CRF compared to girls. However, there were no significant gender differences in PC.

**Figure 1:**
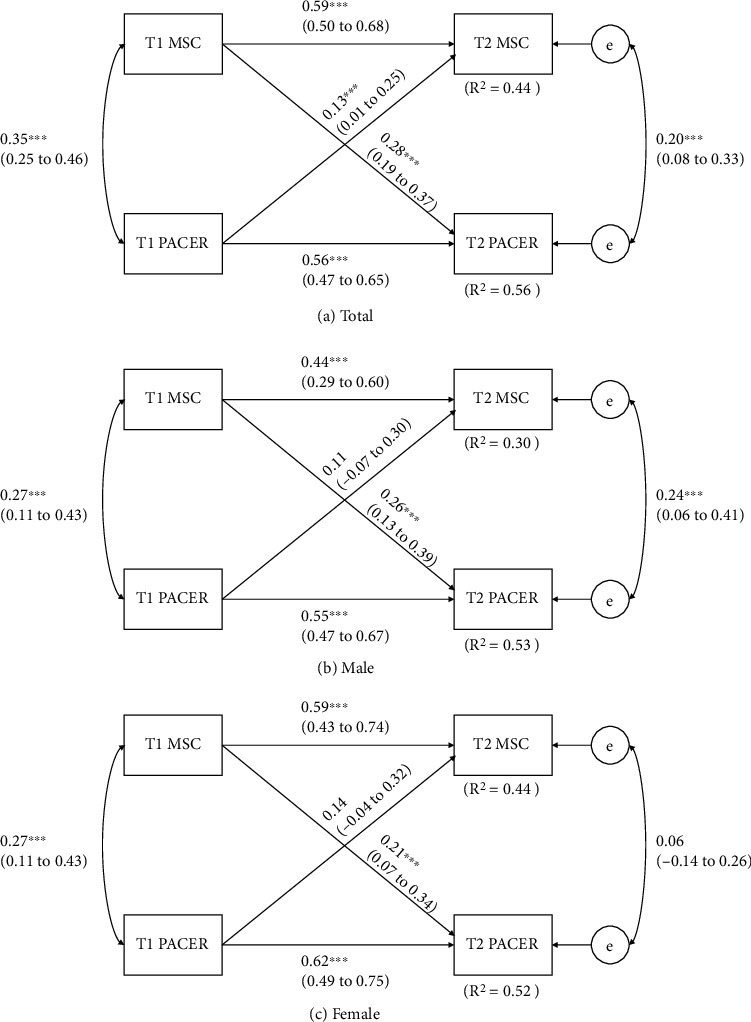
Cross-lagged model of the bidirectional relationships between MSC and CRF before and 8 months. T1 = baseline time, before 8 months; T2 = follow-up time, after 8 months. Path coefficients are standardized with 95% confidence intervals. ^∗^Statistically significant path coefficient (*p* < 0.05).

The results from model fit test indicated that the data fit all six age- and BMI-adjusted cross-lagged panel models well (CFI = 1.00; RMSEA = 0.00).  Figure 1 shows the results of the cross-lagged models between MSC and CRF. Overall, the model explained approximately 44% and 56% (*R*^2^ = 0.44 and 0.56) of variances of T2 MSC and CRF, respectively. In gender-specific models, 30% to 44% (*R*^2^ = 0.30-0.44) and 52% to 53% (*R*^2^ = 0.52-0.53) of the variation in MSC and CRF can be explained, respectively. That is, in the total sample, significant bidirectional relationship was observed between MSC and CRF: T1 MSC significantly correlated with T2 MSC (*p* < 0.01) and T2 CRF (*p* < 0.01); T1 CRF significantly predicted T2 CRF (*p* < 0.01) and T2 MSC (*p* = 0.04); and there were significant correlation between T1 MSC and T1 CRF (*p* < 0.01) as well as T2 MSC and T2 CRF (*p* < 0.01). These relationships shown in the total sample were also held in gender-specific cross-lagged models. Yet, cross-lagged relationships of T1 CRF and T2 MSC were not significant in both males (*p* = 0.24) and females (*p* = 0.13). Additionally, the significant autoregressive effect of MSC tended to be stronger in females (*β* = 0.59) compared to males (*β* = 0.44).

**Figure 2:**
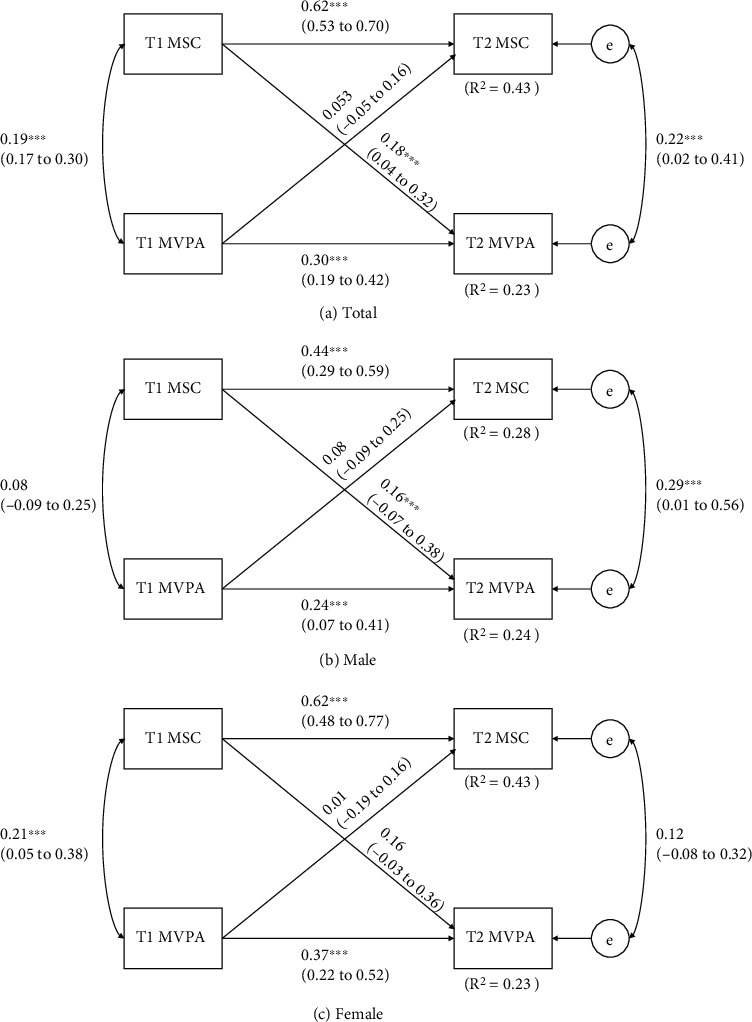
Cross-lagged model of the bidirectional relationships between MSC and MVPA before and after 8 months. T1 = baseline time, before 8 months; T2 = follow-up time, after 8 months. Path coefficients are standardized with 95% confidence intervals. ^∗^Statistically significant path coefficient (*p* < 0.05).

Figure 2 shows the cross-lagged models between MSC and MVPA. A total sample model explained approximately 43% and 23% (*R*^2^ = 0.43 and 0.23) of variances in T2 MSC and MVPA. In gender-stratified models, 28% to 43% (*R*^2^ = 0.28-0.43) and 23% to 24% (*R*^2^ = 0.23-0.24) of the variation in MSC and MVPA can be explained. Overall, no significant bidirectional relationship was observed in the total sample cross-lagged model because significant cross-lagged effect was only observed between T1 MSC and T2 MVPA (*p* < 0.05). Both autoregressive pathways presented significance in T1 and T2 MSC (*p* < 0.01) as well as T1 and T2 MVPA (*p* < 0.01). Also, significant correlations were detected between MSC and MVPA at both T1 (*p* < 0.01) and T2 (*p* < 0.05). Most relationships observed using the total sample were also observed in cross-lagged models for males and females. In addition, the significant autoregressive relationships in both MSC and MVPA tended to be stronger in females (*β* = 0.62; *β* = 0.37) compared to males (*β* = 0.44; *β* = 0.24).

**Figure 3:**
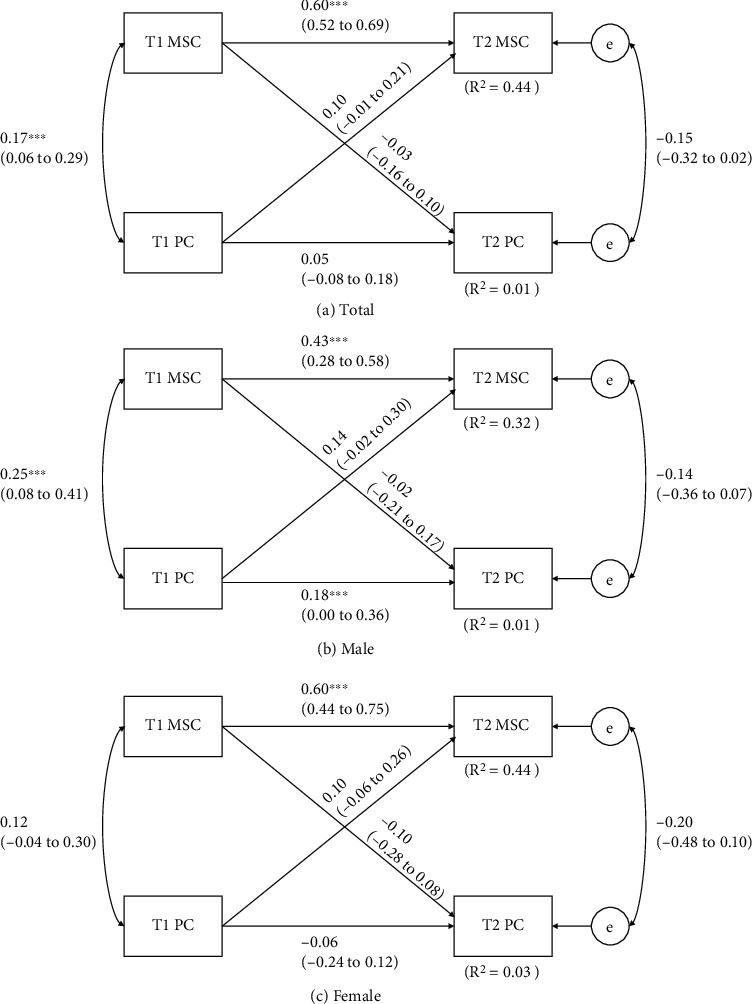
Cross-lagged model of the bidirectional relationships between MSC and PC before and after 8 months. T1 = baseline time, before 8 months; T2 = follow-up time, after 8 months. Path coefficients are standardized with 95% confidence intervals. ^∗^Statistically significant path coefficient (*p* < 0.05).

Figure 3 presents the results of the cross-lagged models between MSC and PC. The model using the total sample explained approximately 44% and 1% (*R*^2^ = 0.44 and 0.01) of variances of T2 MSC and PC. The gender-modified models explained 32% to 44% (*R*^2^ = 0.32-0.44) and 3% to 4% (*R*^2^ = 0.03-0.04) of the variation in MSC and PC, respectively. Cross-lagged effects were not detected in the total sample as the only significant relationships were observed between T1 and T2 MSCs (*p* < 0.01) and between T1 MSC and T1 PC (*p* < 0.01). The significant autoregressive effects of MSC observed using the total sample were held in the gender-modified analyses. Yet, correlation between T1 MSC and T1 PC was statistically insignificant in females only (*p* = 0.13), and significant autoregressive relationship of PC was only observed in males (*p* < 0.05).

**Figure 4:**
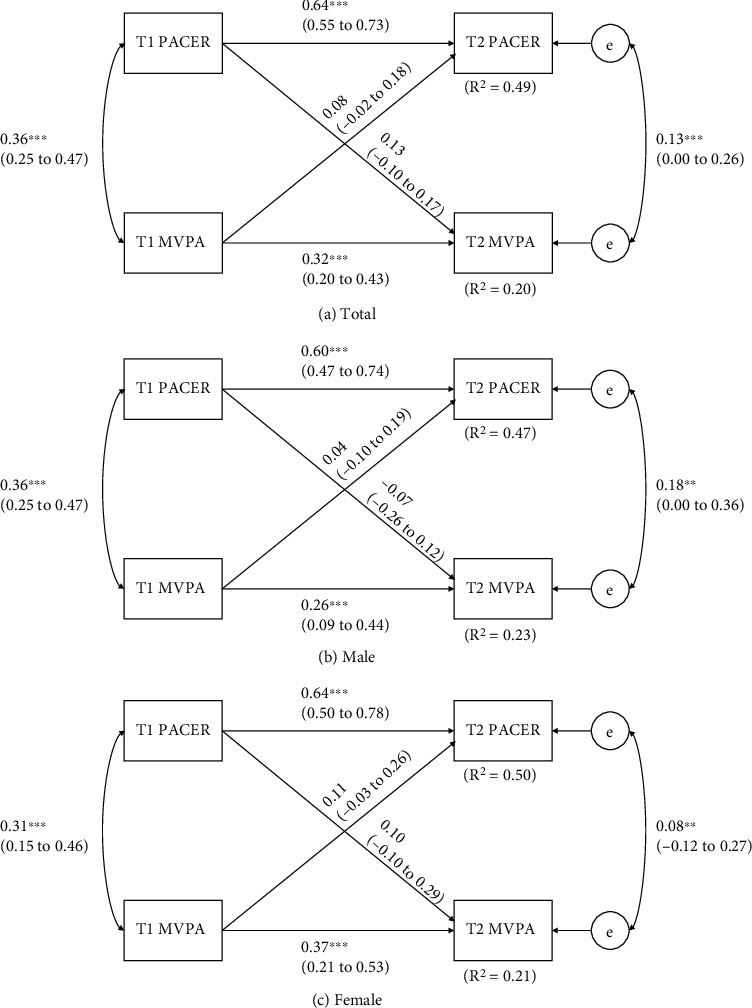
Cross-lagged model of the bidirectional relationships between CRF and MVPA before and after 8 months. T1 = baseline time, before 8 months; T2 = follow-up time, after 8 months. Path coefficients are standardized with 95% confidence intervals. ^∗^Statistically significant path coefficient (*p* < 0.05).

Figure 4 demonstrates the relationship between children's CRF and MVPA by cross-lagged models. Overall, the total sample model explained approximately 49% and 20% (*R*^2^ = 0.49 and 0.20) of variances of T2 CRF and MVPA. In gender-specific models, 47% to 50% (*R*^2^ = 0.47-0.50) and 21% to 23% (*R*^2^ = 0.21-0.23) of the variation in CRF and MVPA can be explained. The results for the total sample cross-lagged model had significant autoregressive effects in both CRF (*p* < 0.01) and MVPA (*p* < 0.01). However, the model did not demonstrate significant cross-lagged effects; thus, there was no bidirectional relationship between CRF and MVPA. Also, there were significant correlations between T1 CRF and T1 MVPA (*p* < 0.01) along with T2 CRF and T2 MVPA (*p* < 0.05). The relationships found for the total sample also were held in the gender-modified analyses.

**Figure 5:**
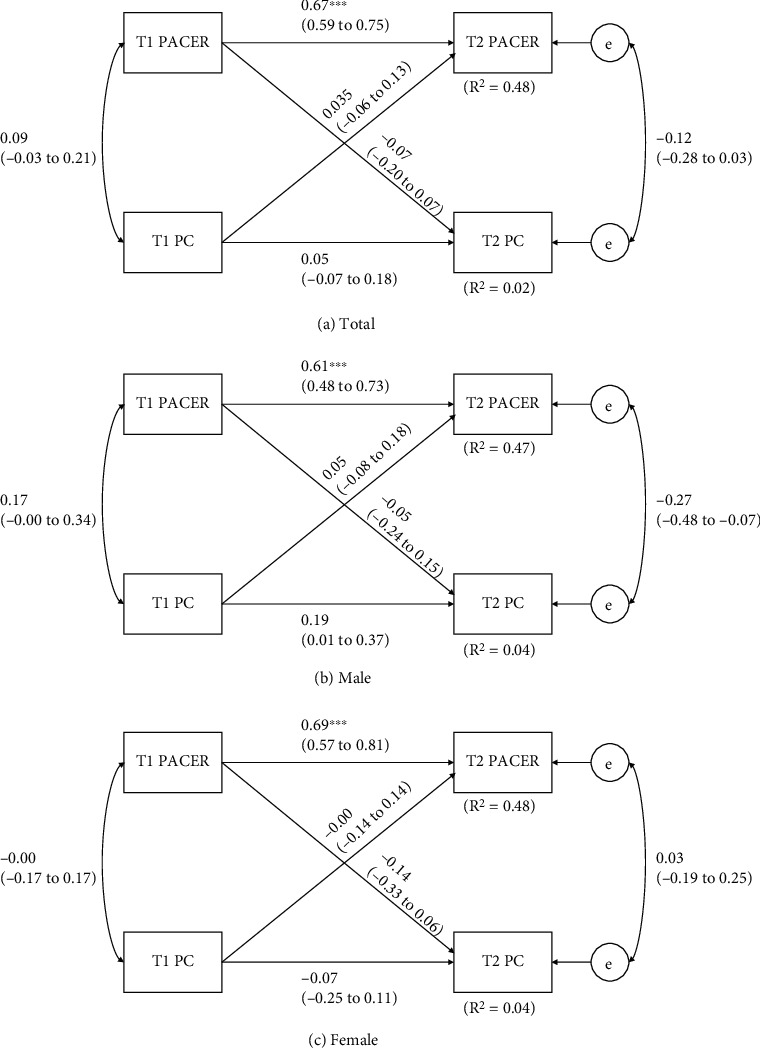
Cross-lagged model of the bidirectional relationships between CRF and PC before and after 8 months. T1 = baseline time, before 8 months; T2 = follow-up time, after 8 months. Path coefficients are standardized with 95% confidence intervals. ^∗^Statistically significant path coefficient (*p* < 0.05).

Figure 5 shows the cross-lagged models between CRF and PC. The model using whole subjects explained approximately 48% and 2% (*R*^2^ = 0.48 and 0.02) of variances of T2 CRF and PC. In gender-stratified models, 47% to 48% (*R*^2^ = 0.47-0.48) and 4% (*R*^2^ = 0.04) of the variation in CRF and PC can be explained. Overall, the only significant patterns of association observed was between T1 CRF and T2 CRF (*p* < 0.01) in all three analyses.

**Figure 6:**
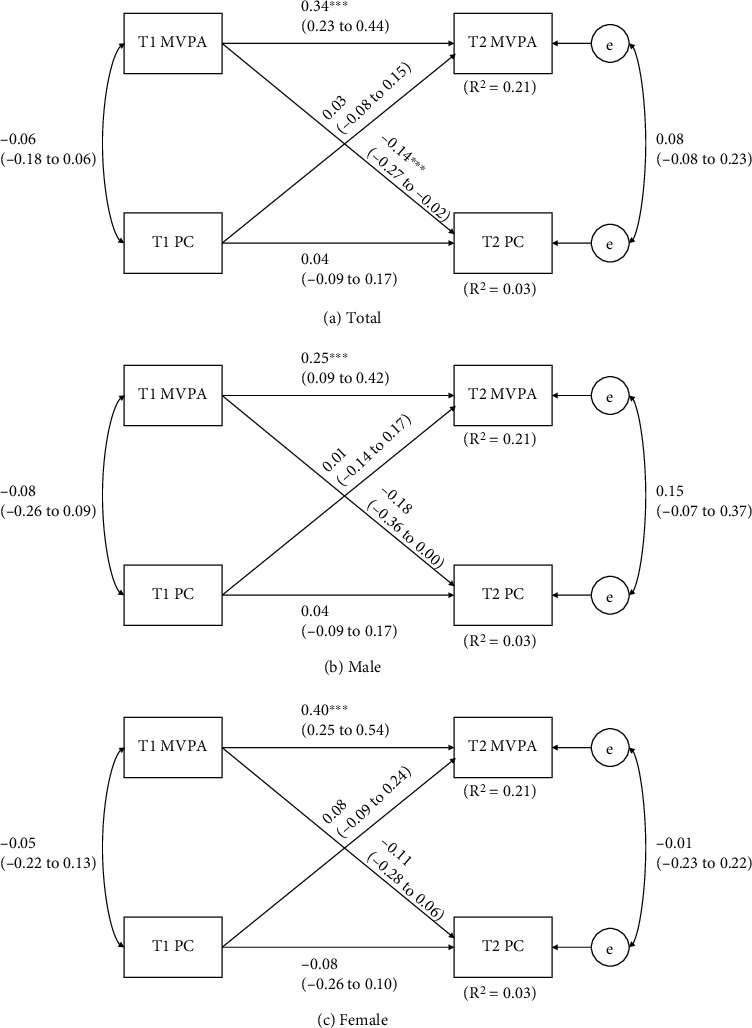
Cross-lagged model of the bidirectional relationships between MVPA and PC before and after 8 months. T1 = baseline time, before 8 months; T2 = follow-up time, after 8 months. Path coefficients are standardized with 95% confidence intervals. ^∗^Statistically significant path coefficient (*p* < 0.05).

Figure 6 demonstrates the results of the relationship between MVPA and PC by cross-lagged models. Total subjects model explained approximately 21% and 3% (*R*^2^ = 0.21 and 0.03) of variances of T2 MVPA and PC. The gender-modified models explained 21% to 23% (*R*^2^ = 0.21-0.23) and 3% to 6% (*R*^2^ = 0.03-0.06) of the variation in MVPA and PC. The results for cross-lagged model using the whole sample had significant and negative cross-lagged effect in T1 MVPA and T2 PC (*β* = −0.14, *p* = 0.03) and significant autoregressive effect in T1 MVPA and T2 MVPA (*p* < 0.01). The observations using the total sample were held in both males and females, with the exception being statistically insignificant cross-lagged effects between T1 MVPA and T2 PC (*p* = 0.05; *p* = 0.20).

## 4. Discussions

This study investigated the bidirectional relationships among elementary children's MS, PC, PA, and CRF over the course of one school year. Among a series of cross-lagged model tests, bidirectional relationship existed between MSC and CRF.

### 4.1. MSC and CRF

Our study results for the total sample support the bidirectional relationship between MSC and CRF. The observation regarding significant predicative relationships between MSC and CRF is in accordance with previous studies supporting MSC could be a good predictor of CRF [9, 14, 39–41] in children and adolescents and vice versa [42]. Indeed, Stodden et al. [14] reported reciprocal relationship between MSC and CRF in a sample of 456 children. Also, Haga [41] found that children with low MSC showed reduced physical fitness than children who had higher MSC. In addition, Cattuzzo et al. [15] in their recent systematic review confirmed a significant and positive relationship between cardiovascular fitness and motor competence in children and adolescents.

### 4.2. MSC and MVPA

Moreover, our findings of total sample suggested that although MSC significantly predicted later MVPA, there was no evidence of cross-lagged effects of MVPA predicting MSC. Hence, a bidirectional relationship between MSC and MVPA was not observed. Yet, our finding of cross-lagged effect between MSC and MVPA in the whole sample is consistent with previous empirical studies which demonstrated that greater MSC was positively associated with vigorous-intensity PA [38, 43–48]. However, none of cross-lagged relationships were observed in gender-modified analyses, which is in line with a recent study conducted by Slykerman et al. [49]. The lack of association between MSC and MVPA in the current study can perhaps be explained by the fact that MSC for children in this study was measured in a total average score of different motor skills. In fact, Slykerman et al. [49] found no association between motor skill variables and MVPA, except for locomotor skill being a significant predictor of MVPA for girls only.

### 4.3. MSC and PC

In terms of MSC and PC, our findings for neither total sample nor gender-modified cross-lagged models presented a bidirectional relationship. These observations are incongruent with previous studies [50–53]. For instance, Robinson [52] reported that children with higher PC exhibit better persistence to master a skill, while children with lower PC easily lose interest in the task at hand. The reason for inconsistent findings from this study could be attributed to children's lack of cognitive skills to distinguish between actual competence and effort [26], leading children to have inflated perceived competence [53]. Also, different MSC measurement tools used in various studies may also explain the inconsistent results [54]. Previous studies used the Test of Gross Motor Development-2nd edition (TGMD-2) [52], whereas the current study used product scores (run, kick, and throw speed and hop and jump distance) to measure MSC.

### 4.4. CRF and MVPA

Additionally, our study results for the cross-lagged model of CRF and MVPA presented autoregressive relationships in both CRF and MVPA, yet bidirectional relationships were not observed in either total sample or sex-specific analyses. Our findings are inconsistent with several previous observations which demonstrated synergetic association between CRF and MVPA [9, 16, 55–60]. Indeed, Stodden et al. [9] suggested health-related physical fitness as a central function in supporting PA behavior. Moreover, Jaakkola et al. [60] demonstrated physical fitness as a significant predictor of later PA engagement. The reason for inconsistent findings is partially attributable to varied measurements of PA and health-related physical fitness across these studies. For instance, compared to our study, previous observations used self-reported PA assessment [60] to capture PA behavior.

### 4.5. CRF and PC

Furthermore, neither total sample nor gender-modified analyses of CRF and PC reported significant bidirectional relationships. The only significant findings observed in the three cross-lagged models were autoregressive effects of CRF and past CRF predicting future CRF. These observations are incongruent with previous studies [61, 62]. A potential reason could be due to divergent ways to assess PC and CRF. For instance, Gao [61] used a shuttle run test to measure children's cardiorespiratory fitness performance and reported that it positively related to their PC and vice versa. Yet, our study measured children's CRF via PACER test.

### 4.6. MVPA and PC

Lastly, the bidirectional relationships between MVPA and PC were not observed in this study. Although our hypothesis was not supported, we observed negative association between T1 MVPA and T2 PC in the whole sample, indicating children with higher MVPA had lower PC after about 8 months. It is possible that children in this study had inflated PC at T2 while their MVPA tended to be higher at the beginning. This cross-lagged effects of MVPA and later PC contrasts previous studies [52, 63, 64]. The conflicting results could be attributable to discrepancy in PA assessments and young children's inflated PC [53] as well as children's different backgrounds of race/ethnicity [54, 65]. For instance, Zeng et al. [54] have shown that children's PC varies by their racial backgrounds.

## 5. Implications and Limitations

The present study's observations shed light on the practical implications of understanding the correlated and mechanisms of PA behaviors among elementary school children. First, based on our findings, educators and health professionals could consider how to adapt interventions to elementary school children, specifically tailored to improving MSC and physical fitness of children, which are important factors in relation to health outcomes in children [14] even in young adults [66]. Additionally, elementary school years are an important target period for behavioral change strategies, as motor skill acquisition in this age cohort may develop and consolidate various motor activities that are necessary to participate in MVPA in later life [67–69]. Hence, our study could contribute to inform the design of effective interventions and strategies, aimed at encouraging young children to be more physically active. Finally, current study targets underserved children who are different from other peers in middle or higher socioeconomic status family regarding physical, psychological, and cognition development. Therefore, our findings for underserved population will assist educators and health professionals in refining the intervention components and reduce health disparities among various demographic sectors.

This study had the following strengths: (1) it has a decent sample size at both T1 and T2; (2) it used a cross-lagged panel model to examine the direction and magnitude of the associations between CRF, MVPA, MSC, and PC. The present study design enabled testing temporal directions of the associations which is a prerequisite for the investigation of causal inference in future studies; and (3) it objectively measured CRF, MVPA, MSC, and PC in school children. However, several study limitations should be addressed: (1) the sample does not represent all and thus may limit the generalizability of the findings; (2) some children may have reached proficiency in their MSC at follow-up test (T2), and thus, there might be a ceiling effect; and (3) the mutual causal effects among the variables could not be established in this study.

In summary, findings of the present study suggested a fully bidirectional relationship between elementary children's MSC and CRF. Other bidirectional relationships among the variables were only partially supported. Educators and health professionals need to emphasize the importance of developing both MSC and CRF to maintain physical health over time. Investigating the mediating role of PC between MVPA and MSC/CRF may be further investigated.

## 6. Conclusion

In conclusion, findings of the present study add the current literature on the roles of dynamic and developmental relationship among MSC, CRF, and PC in contributing to children's PA participation using a cross-lagged model panel approach. Notably, this study is unique in that it is the first to demonstrate the strength of bidirectional relationship between MSC and CRF across one year. Further research should examine children's PA participation involving the bidirectional relationships between MSC, CRF, and PC and how they positively and negatively impact developmental trajectories of PA. Also, future research is needed to focus on bidirectional relationships between types of motor skills (locomotor and object control skills) and multiple health-related physical and psychological factors related to PA.

## Figures and Tables

**Table 1:** Descriptive statistics (mean and standard deviation).

	Total sample (*N* = 261)	Girls (*n* = 134)	Boys (*n* = 127)
Age (years; T1)	8.27 (0.70)	8.29 (0.74)	8.25 (0.66)
Age (years; T2)	10.03 (0.78)^+++^	10.07 (0.80)	9.98 (0.74)
BMI (T1)	18.22 (3.71)	18.21 (3.57)	18.23 (3.92)
BMI (T2)	18.18 (4.77)	18.12 (4.58)	18.25 (4.99)
Total MVPA (T1)	83.38 (42.39)	76.73 (37.57)	92.36 (45.82)^∗∗∗^
Total MVPA (T2)	101.56 (45.94)^+++^	92.45 (35.58)	111.54 (53.45)^∗∗^
Perceived competence (T1)	3.00 (0.6319)	2.99 (0.64)	3.020 (0.59)
Perceived competence (T2)	2.44 (0.58)^+++^	2.44 (0.57)	2.44 (0.58)
Motor skill competence (T1)	50.08 (6.81)	46.47 (3.45)	53.71 (7.27)^∗∗∗^
Motor skill competence (T2)	50.76 (7.16)	47.02 (3.14)	54.35 (8.04)^∗∗∗^
Physical fitness (T1)	18.16 (10.09)	16.31 (8.93)	21.16 (11.01)^∗∗∗^
Physical fitness (T2)	23.56 (12.17)^+++^	20.15 (9.76)	26.83 (13.34)^∗∗∗^

T1 stands for baseline time; T2 stands for follow-up time. After 8 months, ^+^statistical differences between timepoints: ^++^*p* < 0.05 and ^+++^*p* < 0.01; ^∗^statistical differences between gender: ^∗∗^*p* < 0.05 and ^∗∗∗^*p* < 0.01.

## Data Availability

The data that support the findings of this study are not openly available due to ethical concerns and are available from the corresponding author upon reasonable request.
